# Association Between Cardiovascular Risk Factors and the Severity of Coronavirus Disease 2019: Nationwide Epidemiological Study in Korea

**DOI:** 10.3389/fcvm.2021.732518

**Published:** 2021-09-09

**Authors:** Kyoung Ae Kong, Sodam Jung, Mina Yu, Junbeom Park, In Sook Kang

**Affiliations:** ^1^Department of Preventive Medicine, College of Medicine, Ewha Womans University, Seoul, South Korea; ^2^Division of Cardiology, Department of Internal Medicine, Ewha Womans University Mokdong Hospital, College of Medicine, Ewha Womans University, Seoul, South Korea; ^3^Division of Nephrology, Department of Internal Medicine, Ewha Womans University Seoul Hospital, College of Medicine, Ewha Womans University, Seoul, South Korea

**Keywords:** COVID-19, SARS-CoV-2, cardiovascular disease, risk factor, mortality

## Abstract

**Background:** Acute respiratory viral infections can result in cardiovascular involvement, with such patients having a significantly higher mortality rate than those without cardiovascular involvement. Due to the ongoing coronavirus disease 2019 (COVID-19) pandemic, it is important to determine whether cardiovascular risk factors are associated with the severity of COVID-19.

**Methods:** These nationwide data were provided by the Korea Disease Control and Prevention Agency. We defined a patient as having a “critical illness” if they required more than invasive mechanical ventilation and “fatal illness” if they died.

**Results:** Among the total 5,307 patients, 2,136 (40.8%) were male. The critical illness rate was 5.1% (males: 6.7, females: 4.0%) and the fatality rate was 4.54%. The multivariable analysis showed that age ≥60 years, male sex, diabetes mellitus, hypertension, heart failure, chronic kidney disease, cancer, and dementia were independent risk factors for critical illness. The risk scoring model showed the significance of multiple risk factors. Patients with four risk factors; old age (≥60 years), male sex, hypertension, and diabetes mellitus had a more than a 100 times higher risk for severe COVID-19 than those without these risk factors (OR; 95% confidence interval, 104; 45.6–240.6 for critical, 136.2; 52.3–3547.9 for fatal illness).

**Conclusions:** This study demonstrated that cardiovascular risk factors are also significant risk factors for severe COVID-19. In particular, patients who have multiple cardiovascular risk factors are more likely to progress to severe COVID-19. Therefore, early and appropriate treatment of these patients is crucial.

## Introduction

The risk of myocardial infarction is known to be proportional to the severity of an acute respiratory infection (1). Acute viral pneumonia can result in cardiovascular diseases, such as heart failure, acute myocardial infarction, arrhythmia, and myocarditis. Patients with cardiovascular involvement have a significantly higher mortality rate than those without cardiovascular involvement (1–4).

Since the end of 2019, coronavirus disease 2019 (COVID-19) caused by a novel coronavirus, severe acute respiratory syndrome coronavirus 2 (SARS-CoV-2), has spread to more than 200 countries around the world. It has displayed high transmission power, severity, and mortality. The clinical manifestation of COVID-19 is broad, ranging from no symptoms to fever, acute respiratory distress syndrome, multiple organ failure, and death (5). Many countries around the world have been struggling to contain COVID-19, and so far, no definite treatment has been developed. Therefore, it is important to assess the risk factors that affect the severity and fatality of COVID-19.

Excess mortality was reported in the United States during the influenza pandemic. Although the association with secondary infections was not identified, cardiovascular event may have been a contributing factor (1, 6). A recent observational study showed that underlying cardiovascular diseases, such as coronary artery disease, congestive heart failure, and arrhythmia were associated with an increased risk of in-hospital death by COVID-19 (7). Early on in the pandemic, studies in China showed that people with underlying conditions such as hypertension, diabetes mellitus, cerebrovascular disease, or cardiovascular disease were more likely to be admitted to the intensive care unit (5).

Hence, in this study, we focused and analyzed the clinical implications of cardiovascular risk factors and the presence or absence of cardiovascular disease on the outcome and severity of COVID-19. Research on cardiovascular risk factors, including hypertension and diabetes mellitus, which are prevalent in the entire population, and the effect of sex and age on the severity of COVID-19 are not only important to cardiologists, but are significant public health topics. Further, this study can provide important prognostic information for patients.

The previous studies are mostly conducted early on in the pandemic and have limited study populations. The Republic of Korea reduced the spread of COVID-19 by proactively and systematically identifying patients with COVID-19 based on the national health insurance system, which is a single-payer, compulsory subscription system. Accordingly, we have a good basis for analyzing the characteristics of the clinical features of COVID-19 using data from across Korea. Here, we analyzed whether cardiovascular disease and/or the associated risk factors affect the severity of COVID-19 using data provided by the Korea Disease Control and Prevention Agency (KDCA).

## Materials and Methods

### Data Source and Study Population

This is a retrospective cohort study, using nationwide data from the Republic of Korea. Study candidates are the patients with COVID-19 who had been hospitalized, among the patients released from isolation or died as of April 30, 2020. Since the first confirmed case of COVID-19 in Korea on January 19, 2020, the KDCA has actively tracked almost all patients and their contacts in an attempt to control the spread of COVID-19. Further, cumulative statistics are released daily on a public web site (http://ncov.mohw.go.kr/en/) and through the media. The KDCA developed a registry of confirmed COVID-19 cases and provided the anonymized data to select researchers. The data includes only COVID-19 patients that had been released from isolation or died until 30 April 2020. We analyzed the data received from the KDCA via encrypted, remote access.

**Figure 1:**
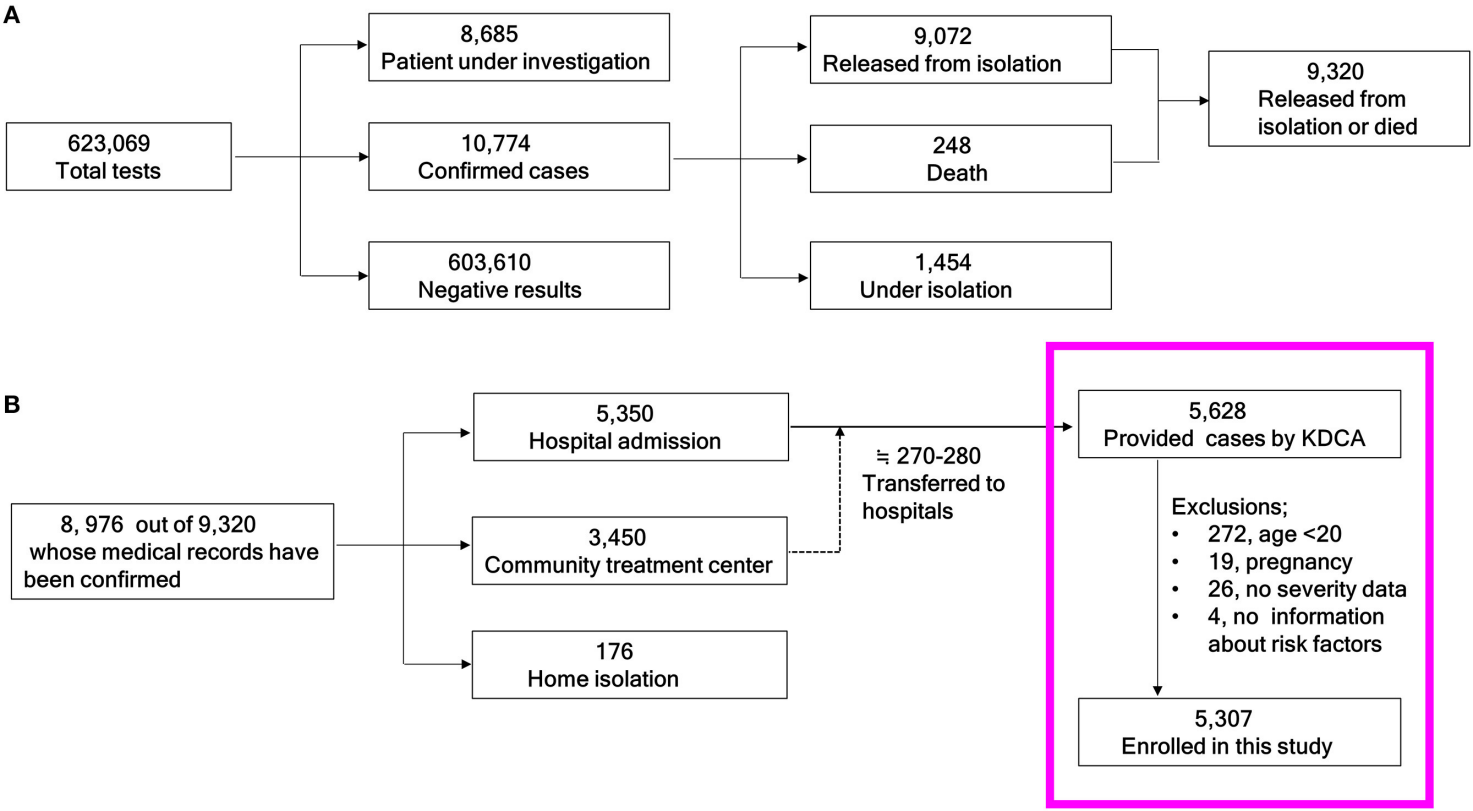
Schema of the study population. **(A)** From the total number of people tested for COVID-19 to the cumulative numbers of those who were released from isolation or died. **(B)** Flow chart of the current study candidates. KDCA, Korea Disease Control and Prevention Agency.

A brief summary of the COVID-19 related quarantine issues in Korea from January 19 to April 30, 2020 is as follows. The approximate total population of the Republic of Korea was 51,780,579 in April 2020 (8). The total number of COVID-19 tests conducted was 623,069 and the number of confirmed cases was 10,774 as of April 30, 2020 (Figure 1A). Among the 10,774 confirmed cases, 1,073 (9.96%) were foreign patients. In total, 9,072 patients had been released from isolation, and 248 had died (fatality rate: 2.73%). Of the 9,072 patients who had been released from isolation, 8,976 had accessible medical records. Of these, 5,350 were hospitalized, 3,450 were admitted to community treatment centers, and 176 were isolated at home. Most of the patients who were isolated at home or community treatment center had asymptomatic or mild disease (9). If disease progressed to moderate or severe condition, they were transferred to hospitals. Of the people who initially entered a community treatment center, approximately 270–280 patients were eventually hospitalized (Figure 1B) (10). The KDCA allowed select researchers temporary access to the anonymized data of 5,628 patients (under granted permission).

A total of 5,628 raw data points corresponding to inpatients from the KDCA were initially reviewed (Figure 1B in the pink box). Finally, 5,307 patients were analyzed after excluding 272 patients under the age of 20 years, 19 pregnant women, 26 without clinical severity information, and four without comorbidity information.

The data included the presence of diabetes mellitus, hypertension, heart failure, chronic heart disease, asthma, chronic obstructive pulmonary disease, chronic kidney disease, malignancy, chronic liver disease, rheumatic/autoimmune disease, and dementia, but did not show the duration of disease and medication history. The co-morbid condition was collected through history taking by medical personnel with questionnaire. There was no available detailed information, disease status and treatment regimens for COVID-19 in the given data.

### Study Definitions

A confirmed case was defined as a patient who had tested positive for SARS-CoV-2 after a real-time reverse transcription-polymerase chain reaction (RT-PCR) test with respiratory specimens: upper respiratory specimens (nasopharyngeal and oropharyngeal swabs), with or without a lower respiratory specimen (sputum), regardless of their clinical manifestations (2). To be released from isolation or discharged, patients had to be: (1) afebrile without symptoms for 10 days and/or (2) have two negative RT-PCR results at least a 24-h interval (11).

Disease severity was defined according to the KDCA and World Health Organization guidelines (11, 12) as follows: level 1, no limitation of daily activities; level 2, limitation of daily activities but no need for oxygen therapy; level 3, oxygen therapy via a nasal cannula; level 4, oxygen therapy via a facial mask; level 5, high-flow supplemental oxygen therapy or non-invasive mechanical ventilation; level 6, needs invasive mechanical ventilation; level 7, multi-organ failure or needs extracorporeal membrane oxygenation (ECMO) therapy; level 8, death. Levels 6–8 were defined as critical illness, whereas 8 was defined as fatal illness. In this study, critical illness is a broader concept that includes fatal illness, and fatal illness refers to a mortality case. The severity evaluation was based on patients with the most severe condition during their hospital stay. For example, fatal illness refers to death of patients regardless of whether they received level 1 or 7 treatment. All fatality cases were made to correspond to level 8. Both critical and fatal illness were considered to be severe COVID-19.

Information on comorbidities was reviewed to determine whether patients had previously been diagnosed with specific comorbidities. Body temperature and body mass index were the initial findings on hospital admission.

### Statistical Analysis

The baseline characteristics of the subjects were described as a frequency and proportion for categorical data. The chi-square test was used to compare the categorical variables. The values of continuous variables were expressed as the median and interquartile range (IQR; Q1, Q3). The Mann-Whitney *U*, or Wilcoxon rank-sum tests were performed for body temperature. Univariate and multivariable logistic regression models were applied to evaluate the risk factors of critical and fatal illness. Age was given as a categorical variable in units of 10 years. There were no critical or death cases reported in the 20–29-year age (20s) group. So, age group was categorized as <40 (20s + 30s) years, 40s, 50s, 60s, 70s, and ≥80 years for logistic regression, and the 60s used as the reference.

Multivariable logistic regression was used to analyze the independent risk of critical and fatal illness after adjusting for several comorbid diseases: diabetes mellitus, hypertension, heart failure, chronic heart disease (other than hypertension and heart failure), bronchial asthma, chronic obstructive lung disease (COPD), chronic kidney disease (CKD), chronic liver disease, rheumatic disease, cancer (excluding cured cases), and dementia.

Utilizing the cardiovascular risk factors, a model for criticality and fatality prediction was made with age ≥60, male sex, medical history of diabetes mellitus, and hypertension as one point each. Theses ranged from a minimum of zero to a maximum of four points.

Next, the criticality and fatality prediction models were analyzed by logistic regression model, odds ratios (ORs) and c-statistics were obtained. The c-statistics were equivalent to the area under the receiver operating characteristic (ROC) curve, based on the predicted probability of the outcomes (the critical or fatal disease) in the logistic regression models with the risk score as independent variable. In this model, each score was treated as binary category of 0 or 1.

For risk score validation, we performed internal validation using bootstrap resampling. To evaluate the performance of compensating overfitting of logistic regression and the risk score model, a total of 1,000 random bootstrap samples were generated for replacement of the original data, and each bootstrap sample size was the same scale as the original data. Then the means and 95% confidence intervals of bootstrap samples were calculated. The c-statistic difference between original data and bootstrap samples was defined as optimism. Optimism-corrected c-statistic can be obtained by subtracting the estimated mean of the optimism estimate value from the c-index in the original sample.

A *P*-value of < 0.05 was considered statistically significance. The statistical analysis was performed using the SAS software (version 9.4, SAS Institute, Cary, NC, USA).

### Ethics Statement

This study was deemed exempt from ethical review and the requirement for informed consent was waived by the Ewha Womans University Mokdong hospital Institutional Review Board (EUMC2020-07-002) because all of the data were fully anonymized and did not include personally identifiable information.

## Results

The baseline demographic and clinical characteristics are presented in Table 1. Among the 5,307 patents, 2,136 (40.8%) were male, the rate of critical illness was 5.1% (male: 6.7, female: 4.0%; *P* < 0.001), and the fatality of the study group was 4.54%. Number of cases is highest in the 20s and 50s, but no critical illness or fatal illness was in the 20s. Meanwhile, critical illness started to rise steeply from the age of 50s, reaching 43.5% in the 80s.

**Table 1:** Baseline characteristics of the study population.

**Characteristics**	**Overall** ** (*n* = 5,307)**	**Critical illness** ** (*n* = 271)**	**Fatal illness** ** (*n* = 241)**	**Incidence (%)**	
	***N* (column %)**	***N* (column %)**	***N* (column %)**	**Critical**	**Fatal**
Age (years)					
20–29	1,104 (20.8)	0 (0)	0 (0)	0	0
30–39	549 (10.3)	3 (1.1)	2 (0.8)	0.5	0.4
40–49	738 (13.9)	2 (0.7)	2 (0.8)	0.3	0.3
50–59	1,141 (21.5)	21 (7.7)	15 (6.2)	1.8	1.3
60-69	906 (17.1)	45 (16.6)	34 (14.1)	5.0	3.8
70–79	545 (10.3)	82 (30.3)	73 (30.3)	15	13.4
≥80	324 (6.1)	118 (43.5)	115 (47.7)	36.4	35.5
Female	3,144 (59.2)	127 (46.9)	114 (47.3)	4	3.6
Male	2,163 (40.8)	144 (53.1)	127 (52.7)	6.7	5.9
Body temperature (°C)Median (Q1,Q3)	36.9 (36.5, 37.3)	37.0(36.6, 37.9)	37.0(36.5, 37.9)		
Cough^*^					
Yes	2,231 (42.0)	92 (33.9)	81 (33.6)	4.1	3.6
No	3,075 (57.9)	179 (66.1)	169 (66.4)	5.8	5.2
Sputum^*^					
Yes	1,549 (29.2)	79 (29.2)	72 (29.9)	5.1	4.6
No	3,757 (70.8)	192 (70.8)	169 (70.1)	5.1	4.5
Sore throat^*^					
Yes	839 (15.8)	14 (5.2)	13 (5.4)	1.7	1.5
No	4,467 (84.2)	257 (94.8)	228 (94.6)	5.8	5.1
Shortness of breath^*^					
Yes	658 (12.4)	134 (49.4)	113 (46.9)	20.4	17.2
No	4,648 (87.6)	137 (50.6)	128 (53.1)	2.9	2.8
Diarrhea^*^					
Yes	504 (9.5)	20 (7.4)	18 (7.5)	4	3.6
No	4,802 (90.5)	251 (92.6)	223 (92.5)	5.2	4.6
Systolic BP^†^					
<120	1,201 (22.6)	66 (24.4)	58 (24.1)	5.5	4.8
120–129	1,076 (20.3)	33 (12.2)	28 (11.6)	3.1	2.6
130–139	1,039 (19.6)	36 (13.3)	32 (13.3)	3.5	3.1
140–159	1,381 (26.0)	77 (28.4)	68 (28.2)	5.6	4.9
≥160	507 (9.6)	41 (15.1)	37 (15.4)	8.1	7.3
BMI (kg/m^2^)^‡^					
<18.5	191 (3.6)	16 (5.9)	16 (6.6)	8.4	8.4
18.5–22.9	1,741 (32.8)	55 (20.3)	46 (19.1)	3.2	2.6
23.0–24.9	1,005 (18.9)	25 (9.2)	20 (8.3)	2.5	2
24.9–29.9	1,011 (19.1)	49 (18.2)	39 (16.2)	4.8	3.9
≥30	193 (3.6)	7 (2.6)	5 (2.1)	3.6	2.6
Diabetes mellitus					
Yes	684 (12.9)	106 (39.1)	98 (40.7)	15.5	14.3
No	4,623 (87.1)	165 (60.9)	143 (59.3)	3.6	3.1
Hypertension					
Yes	1,197 (22.6)	164 (60.5)	144 (59.8)	13.7	12.0
No	4,110 (77.4)	107 (39.5)	97 (40.2)	2.6	2.4
Heart failure					
Yes	59 (1.1)	20 (7.4)	18 (7.5)	33.9	30.5
No	5,248 (98.9)	251 (92.6)	223 (92.5)	4.8	4.2
Chronic heart disease					
Yes	179 (3.4)	29 (10.7)	26 (10.8)	16.2	14.5
No	5,112 (96.3)	242 (89.3)	215 (89.2)	4.7	4.2
Missing	16 (0.3)	0	0	0	0
Asthma					
Yes	126 (2.4)	13 (4.8)	13 (5.4)	10.3	10.3
No	5,181 (97.6)	258 (95.2)	228 (94.6)	5	4.4
COPD					
Yes	38 (0.7)	9 (3.3)	8 (3.3)	23.7	21.1
No	5,269 (99.3)	262 (96.7)	233 (96.7)	5	4.4
Chronic kidney disease					
Yes	55 (1.0)	18 (6.6)	16 (6.6)	32.7	29.1
No	5,252 (99.0)	253 (93.4)	225 (93.4)	4.8	4.3
Cancer					
Yes	145 (2.7)	22 (8.1)	22 (9.1)	15.2	15.2
No	5,162 (97.3)	249 (91.9)	219 (90.9)	4.8	4.2
Chronic liver disease^§^					
Yes	83 (1.6)	7 (2.6)	7 (2.9)	8.4	8.4
No	4,912 (92.6)	264 (91.9)	234 (97.1)	5.4	4.8
Rheumatic disease^||^					
Yes	38 (0.7)	3 (1.1)	3 (1.2)	7.9	7.9
No	4,951 (89.8)	268 (98.9)	238 (98.8)	5.4	4.8
Dementia^¶^					
Yes	224 (4.2)	76 (28.0)	75 (31.1)	33.9	33.5
No	4,768 (89.8)	195 (72.0)	166 (68.9)	4.1	3.5
Severity^**^					
Level 1	4,179 (78.7)	0 (0)	0 (0)	0	0
Level 2	314 (5.9)	0 (0)	0 (0)	0	0
Level 3	468 (8.8)	0 (0)	0 (0)	0	0
Level 4	43 (0.8)	0 (0)	0 (0)	0	0
Level 5	32 (0.6)	0 (0)	0 (0)	0	0
Level 6	19 (0.4)	19 (7.0)	0 (0)	100	0
Level 7	11 (0.2)	11 (4.1)	0 (0)	100	0
Level 8	241 (4.5)	241 (88.9)	241 (100)	100	100

*COPD, chronic obstructive lung disease.*

* *missing n = 1,*

† *missing n = 103,*

‡ *missing n = 1,166,*

§ *missing n = 312,*

|| *missing n = 318,*

¶ *missing n = 315,*

** *Severity level 1, no limitation of daily activities; level 2, limitation of daily activities but no need oxygen therapy; level 3, oxygen therapy via nasal cannula; level 4, oxygen therapy via facial mask; level 5, high-flow supplemental oxygen therapy or non-invasive mechanical ventilation; level 6, the need for invasive mechanical ventilation; level 7, multi-organ failure or the need for extracorporeal membrane oxygenation therapy; level 8, death*.

Clinical symptoms including cough, sputum, sore throat and diarrhea did not differ according to severity, but shortness of breath was more frequently reported in the critical illness than in the non-critical illness (20.4 vs. 2.9%) patients. Patients with systolic blood pressures of <120 or ≥140 mmHg were more likely to be critically ill than those with a systolic blood pressure between 120 and 140 mmHg. Underweight patients with a body mass index of <18.5 kg/m^2^ also showed a higher in the critical illness than in the non-critical illness patients.

Patients with chronic diseases (diabetes mellitus, hypertension, CKD), cardiovascular diseases (heart failure, chronic stable heart disease), respiratory disorder (asthma, COPD), cancer, and/or dementia presented higher rate of critical illness than those without. No significant difference in severity was seen between patients with chronic liver disease or rheumatic disorder and those without. Among the 271 critically ill patients, 19 survived with invasive ventilation and 11 ECMO, whereas the remaining 241 did not survived.

**Figure 2:**
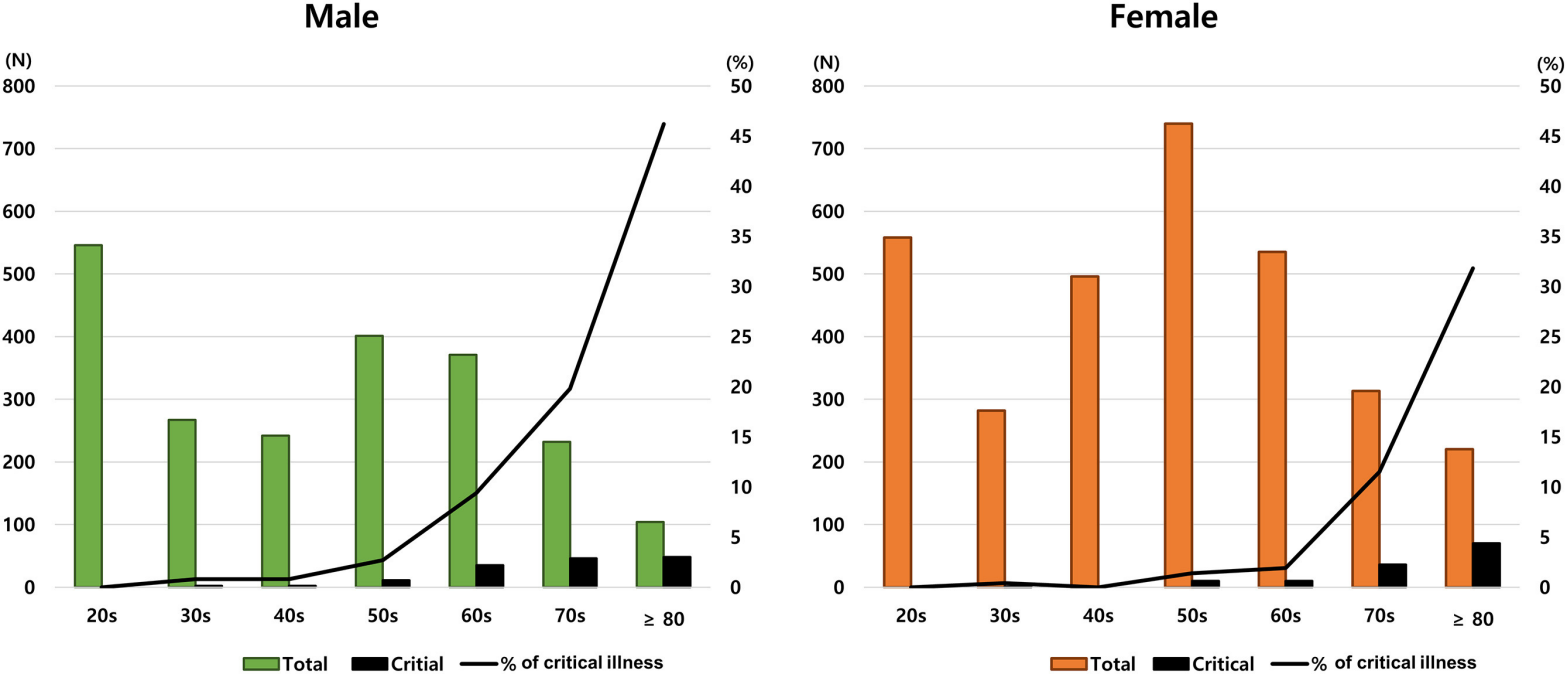
Proportion of critical illness according to age and sex.

Figure 2 shows the disease frequency and critical illness ratio according to sex and age. There were some differences between males and females in the distribution of disease and severity according to age. For example, despite the high frequency of COVID-19 in patients in their 20s, no cases of critical illness were found. In addition, the rate of critical illness increased significantly with age, but this characteristic was more prominent in males.

Logistic analyses were performed to evaluate the risk factors for critical and fatal illness (Table 2). In the univariate analyses of Table 2, all variables were significantly related to both critical and fatal illness except for chronic liver disease and rheumatic disease. Importantly, age was an important risk factor, with those aged <50 years having less risk. Those age ≥70 years had sharply increased ORs. In particular, in patients aged ≥80 years, heart failure, CKD, and dementia had a high OR above 7.0. In the multivariable model 1 in Table 2, asthma and COPD lost their significance for critical and fatal illnesses. Heart failure showed a decreased odds ratio as 2.13 (1.12–4.05) for critical illness and lost the significance of 1.94 (0.99–3.77) for fatal illness.

**Table 2:** Logistic analyses and c-statistics for critical and fatal illness.

	**Critical illness** **Odds ratio (95% CI)**			**Fatal illness** **Odds ratio (95% CI)**		
	**Univariate**	**Multivariable**		**Univariate**	**Multivariable**	
		**Model 1**	**Model 2**		**Model 1**	**Model 2**
**Age (years)**						
<40^*^	0.04 (0.01–0.11)	0.05 (0.02–0.18)	0.05 (0.01–0.15)	0.03 (0.01–0.13)	0.05 (0.01–0.21)	0.04 (0.01–0.17)
40s	0.05 (0.01–0.22)	0.08 (0.02–0.32)	0.07 (0.02–0.29)	0.07 (0.02–0.29)	0.11 (0.03–0.44)	0.09 (0.02–0.39)
50s	0.36 (0.21–0.61)	0.45 (0.26–4.16)	0.43 (0.25–0.73)	0.34 (0.19–0.63)	0.43 (0.23–0.80)	0.41 (0.22–0.75)
60s	1 (reference)	1 (reference)	1 (reference)	1 (reference)	1 (reference)	1 (reference)
70s	3.39 (2.32–4.96)	2.80 (1.89–4.16)	3.10 (2.11–4.58)	3.97 (2.60–6.05)	3.29 (2.13–5.10)	3.68 (2.39–5.65)
≥80	10.96 (7.53–15.96)	7.18 (4.65–11.07)	11.66 (7.87–17.27)	14.11 (9.35–21.29)	9.40 (5.87–15.05)	15.51 (10.09–23.83)
**Male (vs. Female)**	1.89 (1.48–2.42)	2.47 (1.85–3.31)	2.35 (1.77–3.11)	1.85 (1.42–2.40)	2.51 (1.84–3.43)	2.34 (1.73–3.16)
**Diabetes mellitus**	3.80 (2.93–4.92)	1.84 (1.36–2.50)	1.89 (1.40–2.55)	4.02 (3.06–5.28)	2.07 (1.50–2.87)	2.09 (1.52–2.86)
**Hypertension**	4.35 (3.37–5.6)	1.49 (1.10–2.01)	1.49 (1.11–2.00)	4.14 (3.17–5.41)	1.30 (0.94–1.79)	1.29 (0.94–1.77)
**Heart failure**	7.96 (4.57–13.85)	2.13 (1.12–4.05)		7.72 (4.36–13.66)	1.94 (0.99–3.77)	
**CHD**	3.10 (2.04–4.71)	2.13 (1.12–4.05)		3.08 (1.99–4.78)	1.02 (0.60–1.72)	
**Asthma**	2.01 (1.11–3.64)	1.43 (0.70–2.92)		2.30 (1.27–4.16)	1.71 (0.83–3.53)	
**COPD**	4.62 (2.17–9.87)	1.23 (0.48–3.16)		4.50 (2.04–9.93)	1.04 (0.38–2.83)	
**CKD**	7.93 (4.43–14.19)	2.75 (1.33–5.72)		7.54 (4.13–13.77)	2.54 (1.19–5.43)	
**Cancer**	2.83 (1.77–4.54)	2.41 (1.38–4.20)		3.24 (2.02–5.22)	2.89 (1.64–5.10)	
**CLD**	1.37 (0.62–3.00)	1.10 (0.46–2.68)		1.55 (0.71–3.42)	1.32 (0.54–3.23)	
**Rheumatic disease**	1.18 (0.36–3.86)	1.91 (0.52–6.99)		1.34 (0.41–4.39)	2.38 (0.64–8.82)	
**Dementia**	9.42 (6.90–12.87)	2.32 (1.57–3.42)		10.94 (7.96–15.03)	2.58 (1.74–3.84)	
Bootstrap^†^		0.908 (0.891–0.923)	0.903 (0.886–0.919)		0.920 (0.904–0.934)	0.913 (0.896–0.928)
Original, Corrected^‡^		0.905, 0.899	0.902, 0.900		0.917, 0.912	0.912, 0.910

<40* *, 20–39 years; CHD, chronic heart disease; COPD, chronic obstructive lung disease; CKD, chronic kidney disease; CLD, chronic liver disease.*

† *Mean of c-statistics (and 95% confidence interval) of bootstrap samples;*

‡ *C-statistics of original data, the optimism corrected c-statistics*.

In addition, we performed multivariable analyses with four major cardiovascular risk factors (model 2). The results showed similar ORs with model 1 in Table 2. However, ORs of those with age ≥80 was markedly elevated and hypertension lost statistical power of 1.29 (0.94–1.77) for fatal illness.

Model 1 and model 2 showed good performance for prediction of critical illness (original c-statistics, 0.905 and 0.902; optimism-corrected c-statistics, 0.899 and 0.900) and fatal illness (0.917 and 0.912; 0.912 and 0.910). Interestingly, model 2 showed excellent performance similar to model 1. Furthermore, all the values of model 2; bootstrap, original, and corrected, c-statistics showed ≥0.9 for critical and fatal illness.

**Figure 3:**
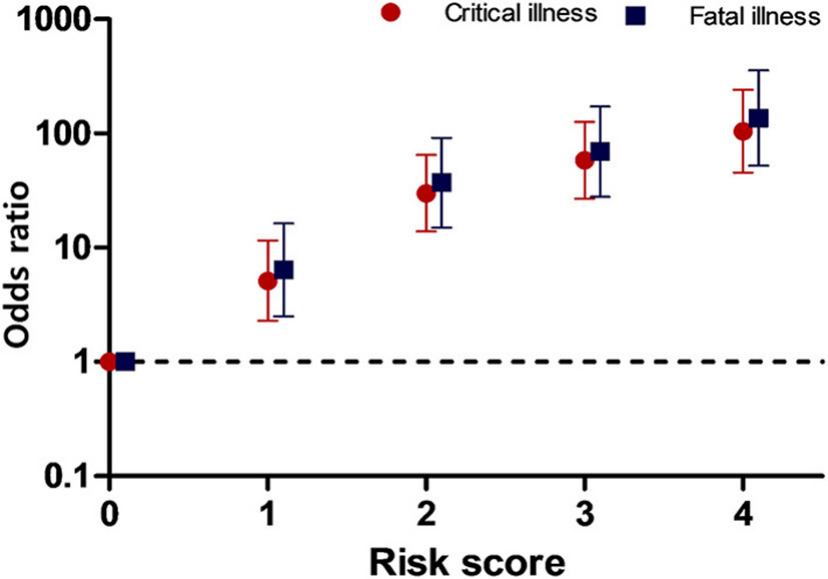
Odds ratios for critical and fatal illness according to the risk score. The scores represent the number of risk factors.

According to the result of model 2, we calculated risk score with simplified four variables: age ≥60 years, male sex, diabetes mellitus, and hypertension, which are also known to be cardiovascular risk factors. Each one risk factor was calculated as one point. Figure 3 shows that the OR for the disease severity increased as the number of risk scores increased relative to the zero point. The ORs (95% confidence interval) for critical and fatal illness were as followings: score 1; 5.1 (2.3–11.5) and 6.4 (2.5–16.3), score 2; 29.9 (13.9–64.7) and 37.2 (15.0–91.9), score 3; 58.4 (36.9–12.8) and 69.2 (27.9–171.8), and 4; 104 (45.6–240.6), and 136.2 (52.3–354.9), respectively. This risk scoring model showed good model fitness (original c = 0.8300 and 0.8321, corrected c = 0.8303 and 0.8324) for critical and fatal illness, respectively.

## Discussion

This study demonstrated that cardiovascular risk factors are also significant risk factors for severe COVID-19. In particular, age ≥60 years was shown to be a strong risk factor; the risk of severe COVID-19 significantly increased by 10 times in those aged ≥80 years compared to those in their 60s. Similar to heart disease, male had a higher risk, and more than twice the odds ratio for critical COVID-19 than female. Hypertension was a risk factor for critical illness rather than fatal illness which was same to heart failure. Additionally, dementia and cancer were found to be poor prognostic factors. Respiratory diseases, such as asthma and COPD were not found to be significant risk factors. Regarding the risk score model, the risk of critical or fatal illness increased sharply according to every increase in score compared to those without risk factors (risk score zero). Therefore, the more risk factors a patient has, the greater their likelihood of progressing to severe COVID-19.

Cardiovascular risk factors are known as smoking, hypertension, diabetes, obesity, physical inactivity, age, male sex (13, 14). However, the data we have only contain diabetes, hypertension, age, sex among the cardiovascular risk factors. Therefore, the risk score was calculated only for the risk factors included in the data. Nonetheless, one interesting thing about this study is that prediction performance of model 2, which included four cardiovascular risk factors (age; six grouped, sex, DM, hypertension), showed as good as that of model 1, which was included 13 variables. When the age groups were simplified as binary group based on the age of 60, the prediction performance was decreased from 0.900 to 0.830 (optimism corrected c) for critical illness. However, which was also good performance. The purpose of this study was to show an association between CV risk factors and the severe COVID-19, rather than developing a new scoring system for predicting severe COVID-19. Through this, we aimed to bring health care providers and patients themselves to have attention of the deleterious effects of multiple CV risk factors in the COVID-19 pandemic era. We intentionally simplified the scoring system as much as possible and included well-known highly prevalence disease.

Previous studies have shown an association between the Middle East respiratory syndrome (MERS) and SARS with acute myocarditis, myocardial infarction, and heart failure as well as a relationship of COVID-19 and myocardial injury (2, 3, 15). These viral infections are all caused by CoV. Furthermore, SARS-CoV-2 has similar pathogenicity to MERS-CoV, which can induce damage to the cardiovascular system, and as a result, can increase the difficulty and complexity of patient treatment (16). There are two implications for this. First is the importance of comorbidities on the prognosis of viral infection. In particular, hypertension and diabetes have been reported as common comorbidities in COVID-19, SARS, and MERS, especially among those with more severe disease (5, 17, 18). In a cohort of 138 hospitalized patients with COVID-19, the reported rate of hypertension was 31% (58% in patients requiring intensive care), and diabetes was 10% (22% in patients requiring intensive care) (5). In the current study, 22.6% had hypertension (60.5% in those with critical illness), 12.9% had diabetes mellitus (39.1% in those with critical illness). Second, it is important to determine whether myocardial damage occurs during viral infection. Data from China showed that elevated level of cardiac biomarker-troponin T was related to increased mortality of patients with COVID-19 regardless of cardiovascular disease (4), and almost 12% of patients without known cardiovascular disease had elevated troponin levels or experienced cardiac arrest during hospitalization (19). We suggest, these results that elevated troponin in other studies is associated with mortality may indirectly explain the mechanism of disease severity and mortality in our study.

Potential mechanisms for the association between acute viral infection and increased myocardial damage are as follows: (1) type 1 myocardial infarction, which is caused by atherosclerotic plaque rupture or coronary thrombosis related acute inflammation; (2) type 2 myocardial infarction, which is related to the mismatch of oxygen demand and supply, and (3) direct effect of the virus and inflammation on the cardiac cells (1, 16, 20). A cardiac metabolic mismatch may be induced by the aggravation of coronary artery stenosis by toxin-mediated vasoconstriction in individuals who already have coronary artery stenosis due to chronic atherosclerotic plaques, particularly in the elderly (1). The current study showed that cardiovascular risk factors are also risk factors for severe COVID-19. However, data regarding cardiac biomarkers, which can evaluate myocardial damage, was not available. Therefore, it can only be presumed that the poor prognosis of patients with multiple cardiovascular risk factors is related to myocardial damage.

Previous studies have shown that elevated cardiac troponin and pro-brain natriuretic peptide are each independently associated with poor outcomes in patients with COVID-19 patients (4, 21). However, there is scarce evidence as to which patients are associated with elevated cardiac biomarkers. The COVID-19 pandemic is still driven by virus mutations, and it is a high possibility that a subsequent global pandemic will be repeated by various respiratory viruses. Therefore, research on COVID-19 and the cardiovascular system should be continued to improve patients' prognoses. Through this study, we identified that patients with multiple CV risk factors are associated with severe COVID-19. Through future follow-up studies, it is important to investigate whether the risk score model/multiple CV risk factors in this study is associated with the proportional increase of cardiac troponin or pro-brain natriuretic peptide or new-onset atrial fibrillation which reflecting cardiac complications and poor outcome of COVID-19 patients.

This study showed that CKD, cancer, and dementia are also risk factors. Dementia is a disease that is more prevalent in older individuals. However, it was still found to be a significant risk factor even after adjusting for age and other comorbid conditions. Several studies have found that CKD is related to an increased risk of mortality from COVID-19 (5, 12). In studies form Europe and America, the mortality of CKD patients was higher than that of the normal group, and inversely proportional to the glomerular filtration rate (11, 22). There are several reasons as to why renal dysfunction worsens COVID-19. First, there is a decrease in immune function in uremic patients (23, 24). A previous study showed that in hemodialysis patients infected with COVID-19, the absolute number of natural killer cells is smaller, and the ratio differs from that in COVID-19 patients without dialysis (25). Second, patients with CKD are known to have a higher risk of cardiovascular disease than patients with normal renal function (26). The mortality rate and risk rate from cardiovascular disease are high, and it is considered to be one of the reasons for the high COVID-19 mortality rate in patients with CKD. In this study, we did not have data on the stage of CKD, and whether patients were undergoing dialysis or had previously had a kidney transplant. We did not include CKD in the risk score model due to heterogeneity and these limitations. However, CKD should be considered an important risk factor for severe COVID-19.

In general, patients with an underlying respiratory disease appear to have a poor prognosis for respiratory infections (27). Previous studies have shown that COPD has a significant effect on the prognosis of COVID-19 pneumonia. As yet, this association has not been confirmed in patients with asthma. In previous meta-analyses, COPD was found to increase the risk of severe COVID-19 with an odds ratio of 4.38 and a relative risk of 1.88, compared to those without COPD (28, 29). However, in the present study, COPD and asthma were not found to affect the criticality and fatality of COVID-19. Since the above studies were meta-analyses, there are methodological differences from the current study. In addition, in this study, only 38 (0.7%) patients had COPD, which might have underpowered the relationship. However, 123 (2.4%) patients had asthma in this study, which also showed no relationship. Hence, further research is needed to determine whether respiratory disease is a risk factor for severe COVID-19.

The current study has some limitations. First, since no cardiac biomarkers were available, such as cardiac troponin or pro-brain natriuretic peptide, that can reflect myocardial damage or heart failure, it is unclear as to whether the disease severity associated with cardiovascular risk factors is directly related to actual heart damage. Second, specific disease condition was not available in this study. For example, we could not know whether the result related to chronic heart disease is due to which of ischemic heart disease, valvular heart disease, and cardiomyopathy. In addition, our data did not include information on atrial fibrillation, which was known to be one of the poor prognostic factors associated with COVID-19 (30). There is no information about the stage of CKD, with the data only indicating whether a patient did or did not have CKD. Third, there was no information about the time or duration for the event (for the critical illness) and censored data. Some variables had missing values and we did not replace the missing values. However, the result of multivariable logistic analysis might not be affected by the missing values. There was no significance difference between the result of multivariable logistic analysis with the limited risk factors which were cardiovascular risk factors and heart diseases (Table 2). Further, the missing data did not affect the result of risk score model of Figure 3.

This study has a strong point as nation-wide cohort study which minimizing selection bias. The data is based on the unique single-payer, compulsory subscription system of the Republic of Korea and infectious disease control system integrated by the government through KDCA. Hence, the study could be good reference to explore the situation of a single country and elucidate interracial differences for future investigation. Another novelty of this study is the study population is younger and predominantly females compared to other large series on COVID-19. Since young patients and female patients were included in the analysis, it can be considered to apply to a wider range of populations.

In conclusion, we have described the clinical characteristics and disease severity of hospitalized patients with confirmed COVID-19 in the Republic of Korea, using nationwide data from 5,307 patients. Our results showed that those over 60 years, of the male sex, or those with heart failure, cardiovascular risk factors; hypertension, diabetes mellitus, and CKD have an increased risk of severe COVID-19. Further, the risk scoring model showed the significance of multiple risk factors. Those with four risk factors, old age (≥60 years), male sex, hypertension, and diabetes mellitus, had odds ratio more than 100 of severe COVID-19 than those without these risk factors, although it should be taken into account that it can be statistically exaggerated due to relatively small numbers of patients. In addition, dementia and cancer were also found to be related to severe COVID-19.

## Data Availability Statement

Data cannot be shared publicly for protecting personal information by the Korea Disease Control and Prevention Agency (KDCA). Broad information regarding Korean COVID-19 statistics are released daily on a public web site (http://ncov.mohw.go.kr/en/) and through the media by KCDA. You may contact KDCA and/or the corresponding author for the detailed data in current study.

## Ethics Statement

The studies involving human participants were reviewed and approved by Ewha Womans University Mokdong Hospital Institutional Review. Written informed consent for participation was not required for this study in accordance with the national legislation and the institutional requirements.

## Author Contributions

KK and IK: conceptualization and data curation. KK: formal analysis. KK, SJ, MY, JP, and IK: investigation and validation. SJ and IK: methodology and writing—original draft. SJ: visualization. KK, SJ, and IK: writing—review and editing. All authors contributed to the article and approved the submitted version.

## Conflict of Interest

The authors declare that the research was conducted in the absence of any commercial or financial relationships that could be construed as a potential conflict of interest.

## Publisher's Note

All claims expressed in this article are solely those of the authors and do not necessarily represent those of their affiliated organizations, or those of the publisher, the editors and the reviewers. Any product that may be evaluated in this article, or claim that may be made by its manufacturer, is not guaranteed or endorsed by the publisher.
