# Corrigendum: Association between cardiovascular risk factors and the severity of coronavirus disease 2019: Nationwide epidemiological study in Korea

**DOI:** 10.3389/fcvm.2022.1032843

**Published:** 2022-10-17

**Authors:** Kyoung Ae Kong, Sodam Jung, Mina Yu, Junbeom Park, In Sook Kang

**Affiliations:** ^1^Department of Preventive Medicine, College of Medicine, Ewha Womans University, Seoul, South Korea; ^2^Division of Cardiology, Department of Internal Medicine, Ewha Womans University Mokdong Hospital, College of Medicine, Ewha Womans University, Seoul, South Korea; ^3^Division of Nephrology, Department of Internal Medicine, Ewha Womans University Seoul Hospital, College of Medicine, Ewha Womans University, Seoul, South Korea

**Keywords:** COVID-19, SARS-CoV-2, cardiovascular disease, risk factor, mortality

In the published article, there was an error in “Figure 3.” The maximum risk score in the original figure was mistakenly listed as 5. The correct maximum risk score is 4. The corrected “Figure 3” and its caption appear below.

**Figure 3:**
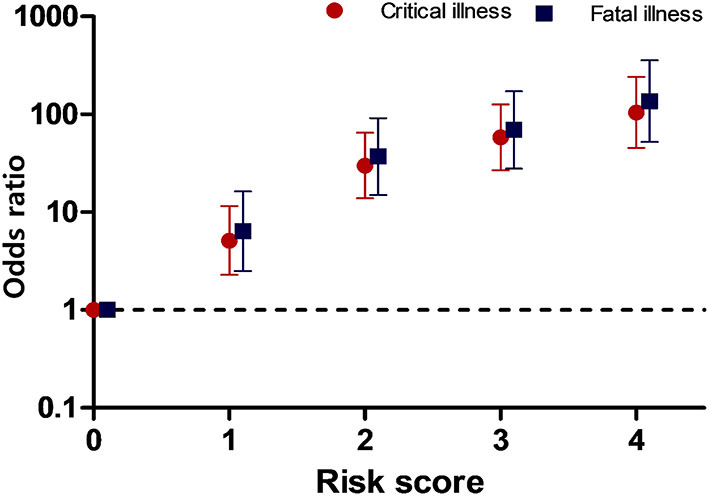
Odds ratios for critical and fatal illness according to the risk score. The scores represent the number of risk factors.

The authors apologize for this error and state that this does not change the scientific conclusions of the article in any way. The original article has been updated.

## Publisher's note

All claims expressed in this article are solely those of the authors and do not necessarily represent those of their affiliated organizations, or those of the publisher, the editors and the reviewers. Any product that may be evaluated in this article, or claim that may be made by its manufacturer, is not guaranteed or endorsed by the publisher.

